# A gait phase prediction model trained on benchmark datasets for evaluating a controller for prosthetic legs

**DOI:** 10.3389/fnbot.2022.1064313

**Published:** 2023-01-05

**Authors:** Minjae Kim, Levi J. Hargrove

**Affiliations:** ^1^Department of Physical Medicine and Rehabilitation, Northwestern University, Chicago, IL, United States; ^2^Regenstein Center for Bionic Medicine, Shirley Ryan AbilityLab, Chicago, IL, United States

**Keywords:** prosthetics, deep learning, continuous gait recognition, benchmark data, gait phase

## Abstract

Powered lower-limb assistive devices, such as prostheses and exoskeletons, are a promising option for helping mobility-impaired individuals regain functional gait. Gait phase prediction plays an important role in controlling these devices and evaluating whether the device generates a gait similar to that of individuals with intact limbs. This study proposes a gait phase prediction method based on a deep neural network (DNN). The long short-term memory (LSTM)-based model predicts a continuous gait phase from the 250 ms history of the vertical load, thigh angle, knee angle, and ankle angle, commonly available on powered lower-limb assistive devices. One unified model was trained using publicly available benchmark datasets containing intact limb gaits for level-ground walking (LGW) and ascending stairs (SA). A phase prediction error of 1.28% for all benchmark datasets was obtained. The model was subsequently applied to a state machine-controlled powered prosthetic leg dataset collected from four individuals with unilateral transfemoral amputation. The gait phase prediction results (a phase prediction error of 5.70%) indicate that the model trained on benchmark data can be used for a system not included in the training dataset with no post-processing, such as model adaptation. Furthermore, it provided information regarding evaluation of the controller: whether the prosthetic leg generated normal gait. In conclusion, the proposed gait phase prediction model will facilitate efficient gait prediction and evaluation of controllers for powered lower-limb assistive devices.

## 1. Introduction

Powered lower-limb assistive devices, such as powered prostheses and exoskeletons, have significant potentials for helping regain functional gait for people with mobility impairments from a variety of causes, including resulting from a spinal cord injury, stroke, or limb amputation.

Generally, these devices are expected to generate joint trajectories to support movements that are based on user intentions. Therefore, a proper understanding of joint kinematics is important for proper control of these devices. For example, prostheses control the powered knee and ankle to restore mobility by analyzing historical information from prior gait strides (Hargrove et al., 2015). Actuating timing of an ankle exoskeleton was controlled for the next stride based on the sensor signal from the previous stride; optimal timing reduced muscle activities and metabolic costs (Galle et al., 2017).

Walking can be represented as a gait cycle. Each gait cycle begins at a heel strike (0%) and ends at the next heel strike of the same leg (100%). The gait cycle can be broken down into a set of periods (e.g., stance and swing), with gait phase representing a specific timing in the gait cycle. Because gait cycle depends on joint kinematics and kinetics, gait phase prediction plays an important role in observing the human state and controlling powered assistive devices. Additionally, the device can be quantitatively evaluated for normal gait generation by analyzing the gait phase.

A variety of methods have been proposed to predict gait phase. Mechanical sensors (Kotiadis et al., 2010; Agostini et al., 2013; Maqbool et al., 2016; Han et al., 2019) (e.g., foot switches embedded in a shoe and inertial sensors on the shank) have been used to determine the gait phase directly. Using a finite-state machine (Lawson et al., 2014) is a popular method for most passive prostheses (Fluit et al., 2019); a state machine sub-divides the gait into a set of discrete phases and defines the transition conditions. A set of surface electromyography sensors on the lower limbs (Yao et al., 2021) were used to predict the discretized gait phase using a neural network having two hidden layers. Since discretization of a gait limits the information during locomotion, continuous gait phase prediction modes have alternatively been proposed. For example, a human-inspired phase variable (Quintero et al., 2017) (e.g., the thigh angle) that uniquely represents the gait cycle in a continuous and monotonically increasing manner was used to predict the gait phase. A discrete wavelet transform-based method (Livolsi et al., 2021) was proposed to predict the continuous gait phase for treadmill walking using hip encoders.

Recently, deep neural network-based (DNN-based) gait phase prediction methods have been proposed. Deep neural networks are highly capable of determining the non-linear relationship between the input and output mapping. The discretized gait phase was predicted using electromyography signals based on a temporal convolutional network (Chen et al., 2022). A recurrent neural network with a shank-mounted inertial measurement unit (IMU) was used to predict the gait phase for controlling an ankle exoskeleton (Seo et al., 2019). In addition, a long short-term memory (LSTM)-based network was proposed to predict the gait phase using a set of wearable sensors, including IMUs and a force sensor at the heel (Lee et al., 2021).

The end-to-end learning capability of DNNs enables gait phase prediction, with multiple ambulation modes showing different gait trajectories. A convolutional neural network-based gait phase estimator (Kang et al., 2021) was proposed to modulate hip exoskeleton assistance for multiple ambulation modes. Gait phases for three different ambulation modes were predicted using a single IMU placed at the shank (Weigand et al., 2020).

Although several studies have successfully demonstrated gait phase prediction, a remaining issue is the lack of efficient adaptation of gait phase prediction methods on a specific system as conventional methods are generally device-specific. In other words, conventional methods were validated using their own hardware. Therefore, a gait phase prediction model trained using a specific system cannot be easily adapted to other systems. Furthermore, training or validating a prediction model for a specific system is time-consuming and burdensome, particularly when targeting mobility-limited patients.

To address this issue, in this study, we propose prediction of continuous gait phase using a DNN. The primary objective was to eliminate the need for device-specific training of gait phase prediction models by using publicly available benchmark datasets (Camargo et al., 2021; Reznick et al., 2021) containing locomotion data of non-disabled individuals. The vertical load (i.e., the weight of users on force plates) and lower limb joint angles [i.e., thigh (induced by hip joint movement), knee, and ankle] were selected as the input data for the proposed gait phase prediction model because these data are commonly available in powered lower-limb assistive devices. Additionally, these data from non-disabled individuals can be collected using various sensor systems.

In conclusion, the proposed method (i) is versatile owing to using sensor data available in generic powered lower-limb assistive devices; (ii) facilitates developing a single unified gait phase prediction model trained on multiple datasets with diverse sensor characteristics; (iii) enables quantitative evaluation of a controller in terms of whether it can generate resembling gait phases of able-bodied individuals.

To validate feasibility, level-ground walking (LGW) and ascending stairs (SA) among various ambulation modes were considered for gait phase prediction because they have distinct differences in gait pattern (Kim et al., 2022).

For demonstration, the proposed method was applied to a prosthetic leg system. The gait phases of a state machine-controlled powered prosthetic leg for transfemoral amputees (Simon et al., 2014) were predicted and evaluated to verify that the proposed method can be used for gait evaluation and has the potential to be used in a system not included in the training datasets.

We hypothesized that the proposed gait phase prediction model could (i) predict the gait phase for a prosthetic leg using a model trained on benchmark dataset, and (ii) it can evaluate the generated gait using the device.

## 2. Methods

The proposed method uses a DNN to predict the continuous gait phase based on the history of the vertical load and joint angles (thigh, knee, and ankle). The gait phase prediction model was trained using benchmark datasets, and its prediction performance across benchmark datasets was evaluated. Then, we collected prosthetic leg data to demonstrate the trained model; four participants with transfemoral amputation then performed in-laboratory LGW and SA using state machine-based impedance controllers at a self-selected speed. Then, the gait phase was predicted from this recorded data. The details of this process are provided below.

### 2.1. Benchmark datasets for training

The proposed method predicts the gait phase using the vertical load and lower limb joint angles (thigh, knee, and ankle). Two benchmark datasets, dataset A (Camargo et al., 2021) and dataset B (Reznick et al., 2021), were selected to train and validate the proposed method; we used the data of LGW on a treadmill and SA.

Datasets A and B were collected from 22 and 10 non-disabled participants, respectively. In both datasets, joint angles from both legs were reconstructed from the motion capture data. In addition, dataset A contains the goniometer data for the joint angles of the right leg. In the case of the LGW, the walking speed ranges from 0.5 to 1.85 m/s for dataset A and from 0.8 to 1.2 m/s for dataset B. The vertical load was measured using force plates embedded in the treadmill. In the case of the SA, we only considered the data from dataset A as dataset B does not provide continuous force plate data for SA. Data from dataset A for SA were collected from four different stair heights of 4, 5, 6, and 7 inches; the vertical load was measured using force plates located on the stairs.

Eight sub-divided datasets were obtained from the benchmark datasets: left/right leg from dataset A, where joint angles were measured using motion capture data (${Am}_{Left}^{LGW}$ and $Am_{Right}^{LGW}$ for LGW, and $Am_{Left}^{SA}$ and $Am_{Right}^{SA}$ for SA); right leg from dataset A, where joint angles were measured using goniometers ($Ag_{Right}^{LGW}$ and $Ag_{Right}^{SA}$); and left/right leg from dataset B ($Bm_{Left}^{LGW}$ and $Bm_{Right}^{LGW}$ for LGW). In conclusion, five LGW datasets (${Am}_{Left}^{LGW}$, ${Am}_{Right}^{LGW}$, $Ag_{Right}^{LGW}$, $Bm_{Left}^{LGW}$, and $Bm_{Right}^{LGW}$) and three SA datasets ($Am_{Left}^{SA}$, $Am_{Right}^{SA}$, and $Ag_{Right}^{SA}$) were extracted. In addition to the sub-divided datasets, all data were included in a dataset, *Alldata*. Thus, there are nine dataset configurations in all. The number of steps collected from each dataset is presented in Table 1.

**Table 1:** Brief description of datasets and the number of steps collected.

**Dataset**	**Description**	**The number of steps**
$Am_{Left}^{LGW}$	Dataset A, LGW, left leg, motion capture	20,650
$Am_{Right}^{LGW}$	Dataset A, LGW, right leg, motion capture	20,685
$Ag_{Right}^{LGW}$	Dataset A, LGW, right leg, goniometer	20,685
$Bm_{Left}^{LGW}$	Dataset B, LGW, left leg, motion capture	1,148
$Bm_{Right}^{LGW}$	Dataset B, LGW, right leg, motion capture	1,286
$Am_{Left}^{SA}$	Dataset A, SA, left leg, motion capture	477
$Am_{Right}^{SA}$	Dataset A, SA, right leg, motion capture	465
$Ag_{Right}^{SA}$	Dataset A, SA, right leg, goniometer	465
*Alldata*	All datasets, all modes, both legs, all sensors	65,861

### 2.2. An open-source bionic leg

The trained gait phase prediction model was validated using an open-source bionic leg (OSL) that contains a powered knee and ankle actuated in the sagittal plane. The details of the OSL system configuration are provided in Azocar et al. (2020). The vertical load and joint angles (thigh, knee, and ankle) were determined using mechanical sensors (i.e., encoders and IMU) embedded in the OSL.

A state machine-based impedance controller (Simon et al., 2014) was used to control the OSL. Each ambulation was sub-divided into four states (i.e., early stance, late stance, early swing, and late swing). Then, the six impedance parameters (i.e., stiffness, damping coefficient, and equilibrium angle for the knee and ankle) were tuned to generate the gait trajectory of an individual state as follows:


(1) $$\begin{array}{l} \tau_{i}=-k_{i}\left(\theta_{i}-\theta_{i}^{eq}\right)-b\overset{•}{\theta_{i}} \end{array}$$

where *i* represents the knee or ankle joint; τ represents the joint torque; *k*, *b*, and θ^*eq*^ denote the stiffness, damping coefficient, and equilibrium angle, respectively; and θ and $\overset{•}{\theta }$ represent the joint angle and velocity, respectively. Impedance parameters were generated every 25 ms. Further details on the state machine and the configuration process are provided in Simon et al. (2014). The impedance parameters were individually adjusted based on user feedback and visual inspections of gait were conducted by a certified prosthetist and licensed therapist.

### 2.3. Participants

Four individuals with unilateral transfemoral amputation (Table 2) participated in this study and performed LGW and SA. All individuals provided written informed consent for the protocol approved by the Northwestern University Institutional Review Board. Users walked with 8-feet parallel bars for LGW, and ascended a 6-step staircase with stair height of 6-inch. We collected as much data as experimental time allowed per user. TF1 and TF2 had each participated in two sessions on separate days; TF3 and TF4 each participated in one session. In each session, we collected an LGW dataset and then an SA dataset. Participants were allowed sufficient rest time during a session under the assistance of a therapist. A total of, 214, 122, 174, and 221 steps were collected for TF1, TF2, TF3, and TF4, respectively. The detailed number of steps per user is given in Table 3.

**Table 2:** Subject demographics.

**User**	**Gender**	**Etiology**	**Height (m)**	**Weight (kg)**
TF1	M	Right sarcoma	1.93	72.57
TF2	M	Left sarcoma	1.93	104
TF3	F	Left sarcoma	1.7	72.5
TF4	F	Right sarcoma	1.65	70.99

**Table 3:** The number of steps per user.

**Mode**	**User**				

	**TF1**	**TF2**	**TF3**	**TF4**	**Total**
LGW	161	104	154	181	600
SA	53	18	20	40	131
Total	214	122	174	221	731

LGW, level-ground walking; SA, ascending stairs.

### 2.4. Gait phase prediction model

The proposed method can be divided into two parts. First, discretized gait events (i.e., toe-off and heel-strike) and phases (i.e., stance and swing phases) are identified from the vertical load based on a peak detection algorithm using z-scores (Brakel, 2014). Although this algorithm is simple and only dependent on the vertical load, continuous gait phase cannot be identified in real-time because gait duration between one heel-strike to the next heel-strike is needed for it. Therefore, to overcome this limitation, the relationship between the continuous gait phase and the sensor data (the joint angles and vertical load) is identified using a DNN.

**Figure 1:**
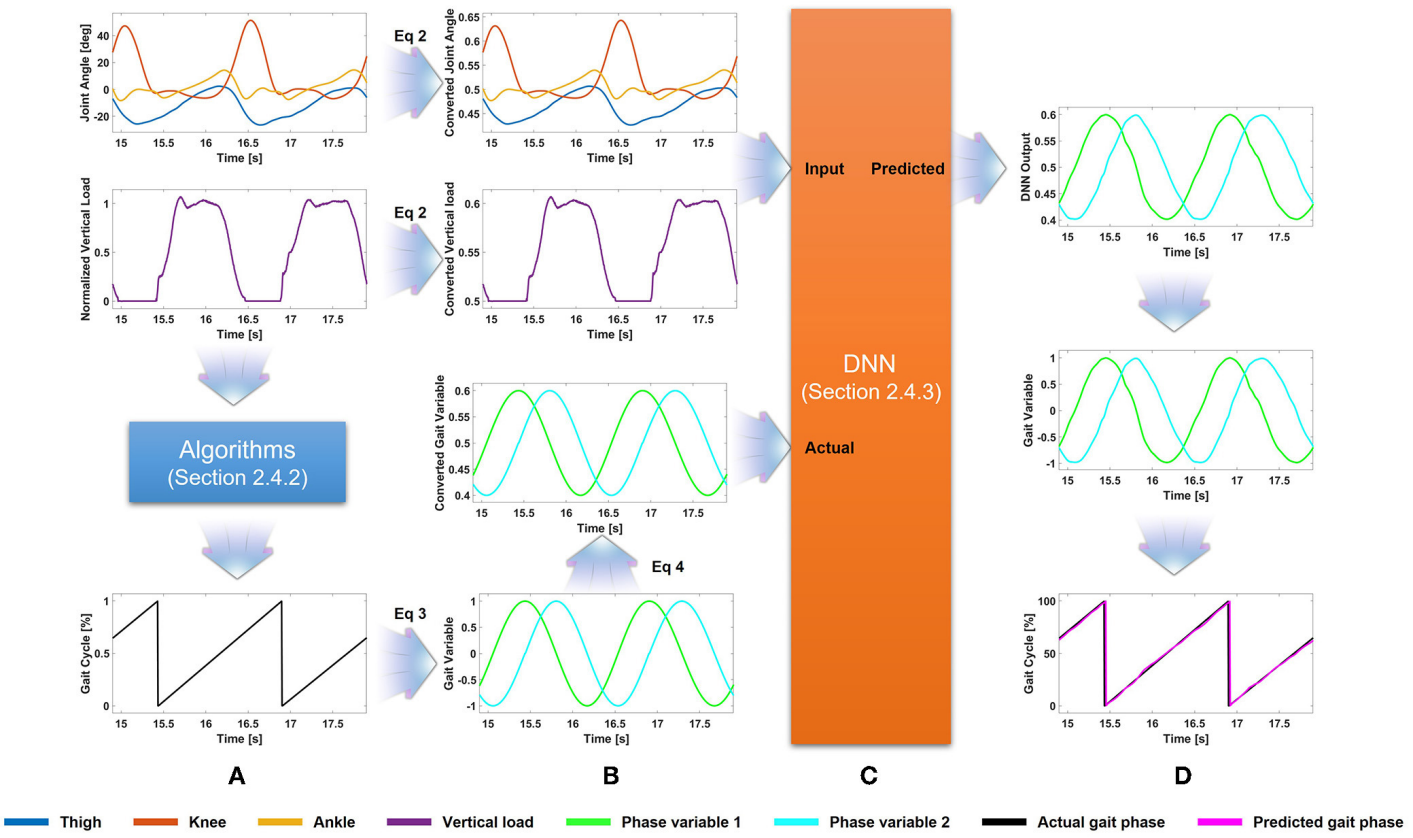
Flowchart of the proposed method. **(A)** Gait phase, extracted from the vertical load, is converted to the 2-D variable. **(B)** The joint angles, vertical load, and gait phase variable are scaled for application to the deep neural network (DNN). **(C)** The scaled (i.e., converted) joint angles and vertical load are used as input for the DNN. **(D)** The DNN output is scaled and converted to the predicted gait phase.

This section describes all procedures for the gait phase prediction. The procedures are shown in Figure 1 using example data. Section 2.4.1 describes data conversion for the DNN application (i.e., data normalization). Section 2.4.2 describes gait segmentation from sensor data and conversion to 2-D variables representing gait phase percentage. Section 2.4.3 describes the architecture of the proposed DNN.

#### 2.4.1. Sensor data conversion

Sensor configurations, including the sampling frequency and sign convention, depend on the experimental setup and hardware specifications. Thus, all sensor configurations were converted to those of the OSL. With respect to the sign convention, thigh extension, knee flexion, and ankle dorsiflexion were defined as positive. Vertical load was normalized by the weight of the user. Since our system recorded data from the OSL every 5 ms, sensor data were downsampled or upsampled to 200 Hz. In addition, owing to differences in sensor configurations, the baseline amplitudes of the joint angles differ. Thus, angular biases were removed to match the baseline with the OSL.

The recommended range of the input data to the DNN is 0 to 1. Therefore, scaling was applied to the sensor data as follows:


(2) $$\begin{array}{l} v_{n}=(\frac{v}{g_{v}}+5)/10\\ g_{v}=\{\begin{array}{cc} 1, & \text{if }v=F_{z}\\ 36, & \text{otherwise} \end{array} \end{array}$$

where *v* denotes the raw data (i.e., the weight-normalized vertical load and joint angles), *v*_*n*_ denotes the data bounded from 0 to 1, *F*_*z*_ denotes the weight-normalized vertical load, and *g*_*v*_ denotes the gain. Thus, for instance, *v*_*n*_ will be 0.5 when *F*_*z*_ is zero (i.e., no weight on the prosthetic leg) and 0.6 when *F*_*z*_ is 1 (i.e., full weight on the prosthetic leg).

#### 2.4.2. Gait phase conversion

The gait events were mathematically extracted (Algorithms 1–3) based on a peak detection algorithm using z-scores (Brakel, 2014). The Supplementary Video S1 shows an example of extraction.

**Algorithm 1:** Gait phase extraction.

Input: $F_{z}\in R^{T}$: *T* time series of the vertical load
Param: *F*_*th*_, *influence*, *lag*, *p*_*hist*_, *filtered*∈*R*^*lag*^
/* Parameter initialization */
1: *F*_*th*_ ← 0.2
2: *lag* ← 8
3: *influence* ← 0.01
4: *p*_*hist*_ ← 5
5: *Filtered*(1:*lag*) ← 0
/* Phase Extraction */
6: for *t* = 1 to *T* **do**
7: [*P*_*ext*_(*t*), *filtered*] = SEGMENT (*F*_*z*_(*t*), *filtered*)
8: end **for**
9: *P*_*output*_ = POST REFINEMENT(*P*_*ext*_)
10: return *P*_*output*_

**Algorithm 2:** Gait segmentation.

1: function SEGMENT(*f*_*z*_, *filtered*)
2: *filtered*(*i*) = *filtered*(*i*+1) for *i* = 1:*lag*−1
3: *f*_*z*_ = 1 if *f*_*z*_>1 and *f*_*z*_=0 if *f*_*z*_ < 0
4: if *f*_*z*_ == mean(*filtered*) **then**
5: *p*_*ext*_ = 0 // swing phase
6: *filtered*(*lag*) = *f*_*z*_
7: else
8: if *f*_*t*_>*F*_*th*_ **then**
9: *p*_*ext*_ = 1 // stance phase
10: else **if** *f*_*t*_≥ mean(*filtered*) **then**
11: // increasing vertical load
12: *p*_*ext*_ = 1 // stance phase
13: else
14: *p*_*ext*_ = 0 // swing phase
15: end **if**
16: *filtered*(*lag*) = *influence*·*f*_*z*_+(1−*influence*)·*filtered*(*lag*−1);
17: end **if**
18: return *p*_*ext*_, *filtered*
19: end **function**

**Algorithm 3:** Post refinement to improve robustness.

1: function POST REFINEMENT(*p*_*ext*_)
2: *p*_*past*_ ← *p*_*ext*_(1)
3: *p*_*current*_ ← −1
4: *count* ← 0
5: *idx*_*bgn*_ ← 1
6: *idx*_*fin*_ ← 0
7: *p*_*re*_(1:*T*) ← −1
8: for *t* = 1 to *T* **do**
9: *p*_*current*_ = *p*_*ext*_(*t*)
10: if *p*_*current*_ ≠ *p*_*past*_ **then**
11: *idx*_*fin*_ = *t*−1
12: if *count*>*p*_*hist*_ **then**
13: *p*_*re*_(*idx*_*bgn*_:*idx*_*fin*_) = *p*_*past*_
14: *idx*_*bgn*_ = *t*
15: *count* = 1
16: else
17: *p*_*re*_(*idx*_*bgn*_:*idx*_*fin*_) = *p*_*current*_
18: *count* = *count*+1
19: end **if**
20: else
21: *count* = *count*+1
22: end **if**
23: *p*_*past*_ = *p*_*current*_
24: end **for**
25: return *p*_*re*_
26: end **function**

Algorithm 1 is applied to the recorded data; it extracts a discretized gait phase (i.e., stance or swing phase), for each time step, *t* based on Algorithm 2.

In every time step, Algorithm 2 subdivides the gait into stance and swing phases according to the vertical load. If the vertical load is higher than *F*_*th*_, the current phase is set to stance. When the vertical load is less than *F*_*th*_, the phase is set to stance if the vertical load tends to increase; and swing if the vertical load tends to decrease. The tendency of the vertical load was determined using the history of *filtered*, which is an output of the peak detection algorithm (Brakel, 2014).

Sensor interference may lead to incorrect extraction; Algorithm 3 improves robustness and refines the extraction. When phase change occurs, if the time length of the previous phase is less than *p*_*hist*_, Algorithm 3 discards this phase change.

Here, all tunable parameters, including *lag*, *influence*, *F*_*th*_, and *p*_*hist*_ were selected heuristically. The values for the peak detection algorithm (*lag* of 8 and *influence* of 0.01) were chosen via visual inspection of the identification results. *F*_*th*_ was set to 0.2 because our state machine-based controller determines the transition from stance phase to swing phase based on this value. *p*_*hist*_ was set to 5, which indicates that phase changes within 25 ms were ignored, because our control frequency of the OSL is 40 Hz.

Using these algorithms, the gait phase percentage was obtained between 0% and 100% in 0.5% increments. As the gait phase percentage increases monotonically from 0% to 100% (i.e., a heel-strike to the next heel-strike), the discontinuities in the percentage of the gait phase from 100% to 0% degrade the prediction performance. Therefore, the gait phase percentage in the Cartesian coordinate system (i.e., 0–100%) was converted into that in the polar coordinate system (Kang et al., 2019) as follows:


(3) $$\begin{array}{c} \theta =2\pi \frac{p_{c}}{100}\\ p_{x}=\cos\theta \\ p_{y}=\sin\theta \end{array}$$

where *p*_*c*_ denotes the gait phase percentage in the Cartesian coordinate system, and *p*_*x*_ and *p*_*y*_ denote those in the polar coordinate system. This gait phase percentage is used as the output of the DNN. Thus, additionally, scaling was applied to the gait phase percentage in the polar coordinate system to normalize the range of the values as follows:


(4) $$\begin{array}{c} P_{i}^{\prime }=\left(p_{i}+5\right)/10 \end{array}$$

where *p*_*i*_ and $p_{i}^{\prime }$ denote the gait phase percentage in the polar coordinate system and its scaled value in the DNN output configuration, respectively. This normalization processing (i.e., Equation 4) may not be necessary. The reason for this scaling is described in Section 4.

#### 2.4.3. A deep neural network

**Figure 2:**
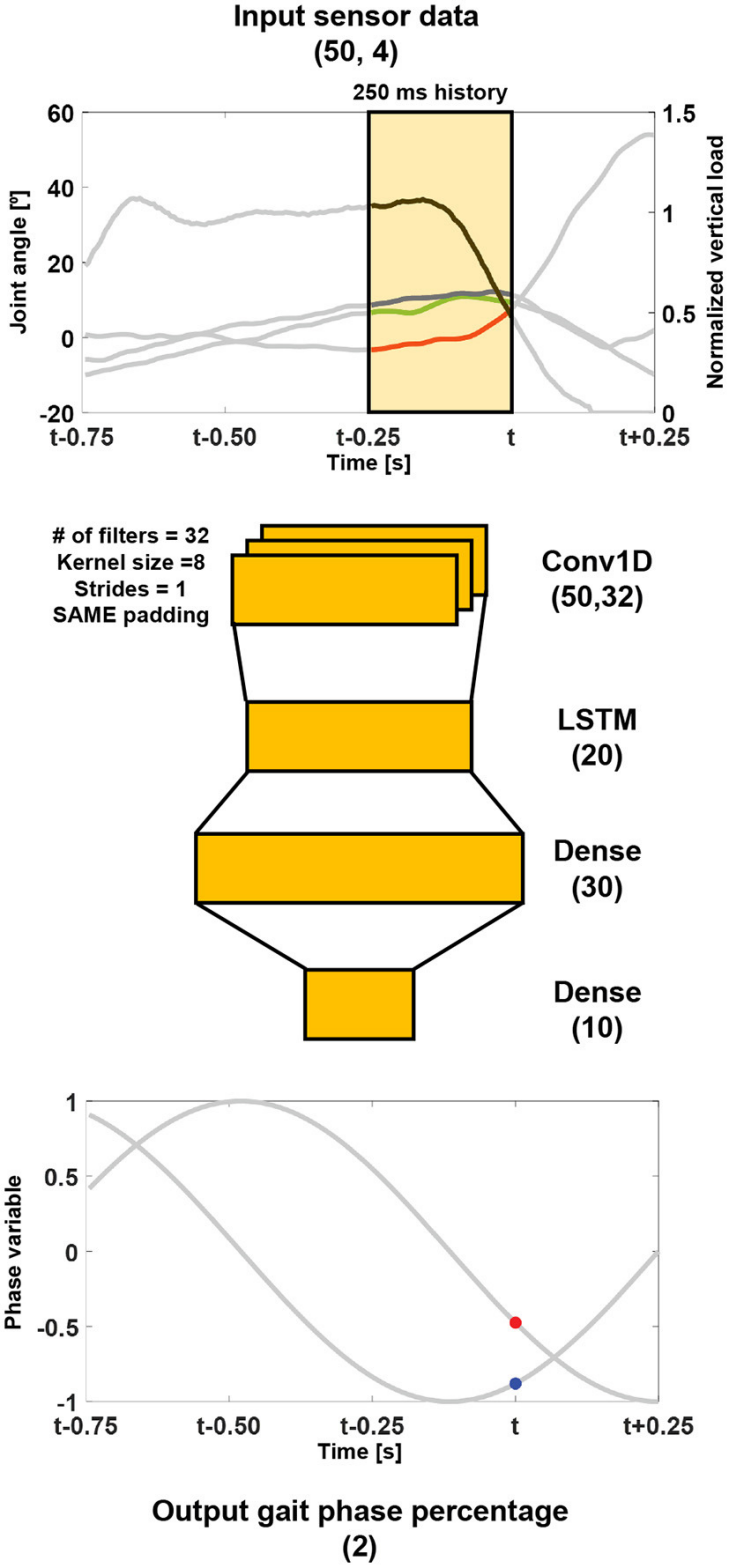
Structure of the proposed network. The gait phase percentage in the polar coordinate system was predicted from a 250 ms history of the vertical load (a black line), and thigh (a green line), knee (a red line), and ankle (a blue line) angles. The numbers in the brackets of the layers represent the shape of each layer. For example, LSTM (*N*) represents the LSTM layer with *N* units.

The structure of the proposed DNN is shown in Figure 2. The input for the network is the 250 ms history of the vertical load, thigh, knee, and ankle angles. The network outputs are the gait phase percentages in the polar coordinate system (i.e., $p_{x}^{\prime }$ and $p_{y}^{\prime }$).

The 1-D convolutional neural network, which leans 32 filters through a kernel size of 8, strides of 1, and the same padding, is connected to the input layer, which is used to capture spatial information from the sensor data. Subsequently, a LSTM layer of 20 units, followed by fully-connected dense layers, is connected to extract temporal information. The network has a total of 6,258 trainable parameters.

The network was trained for 15 epochs with a batch size of 256, the ADAM with a learning rate of 0.001, and the mean squared error as the loss function. At each epoch, the data were shuffled and split into training and validation data in a ratio of 7:3. All the trainable layers had a sigmoid activation function.

The model was trained using TensorFlow (v. 2.7.0, Google) in Python 3.8 on a laptop (Nitro 5 AN517-54-79L1, Acer) with NVIDIA GeForce RTX 3050Ti Laptop GPU and 32 GB DDR4 RAM. The training time for the model trained on *Alldata* (see Table 1) was approximately 210 min.

### 2.5. Data analysis

All nine models were trained using the benchmark datasets; their data configurations are presented in Table 1. The first eight models were trained using eight distinct sub-divided datasets (see Section 2.1). The last model was trained using all benchmark datasets. Each dataset was evaluated using the nine models. Then, gait data from four patients with transfemoral amputation were evaluated using the last model. Any additional processes, such as model tuning, were not performed.

The correlations between datasets were assessed using the Pearson correlation coefficient between the median gait trajectories. The prediction performance was evaluated using the coefficient of determination (*R*^2^) in the polar coordinate system (i.e., $p_{x}^{\prime }$ and $p_{y}^{\prime }$) and phase prediction error for the difference between the actual and predicted gait phases.

## 3. Results

### 3.1. Evaluation of benchmark datasets

**Figure 3:**
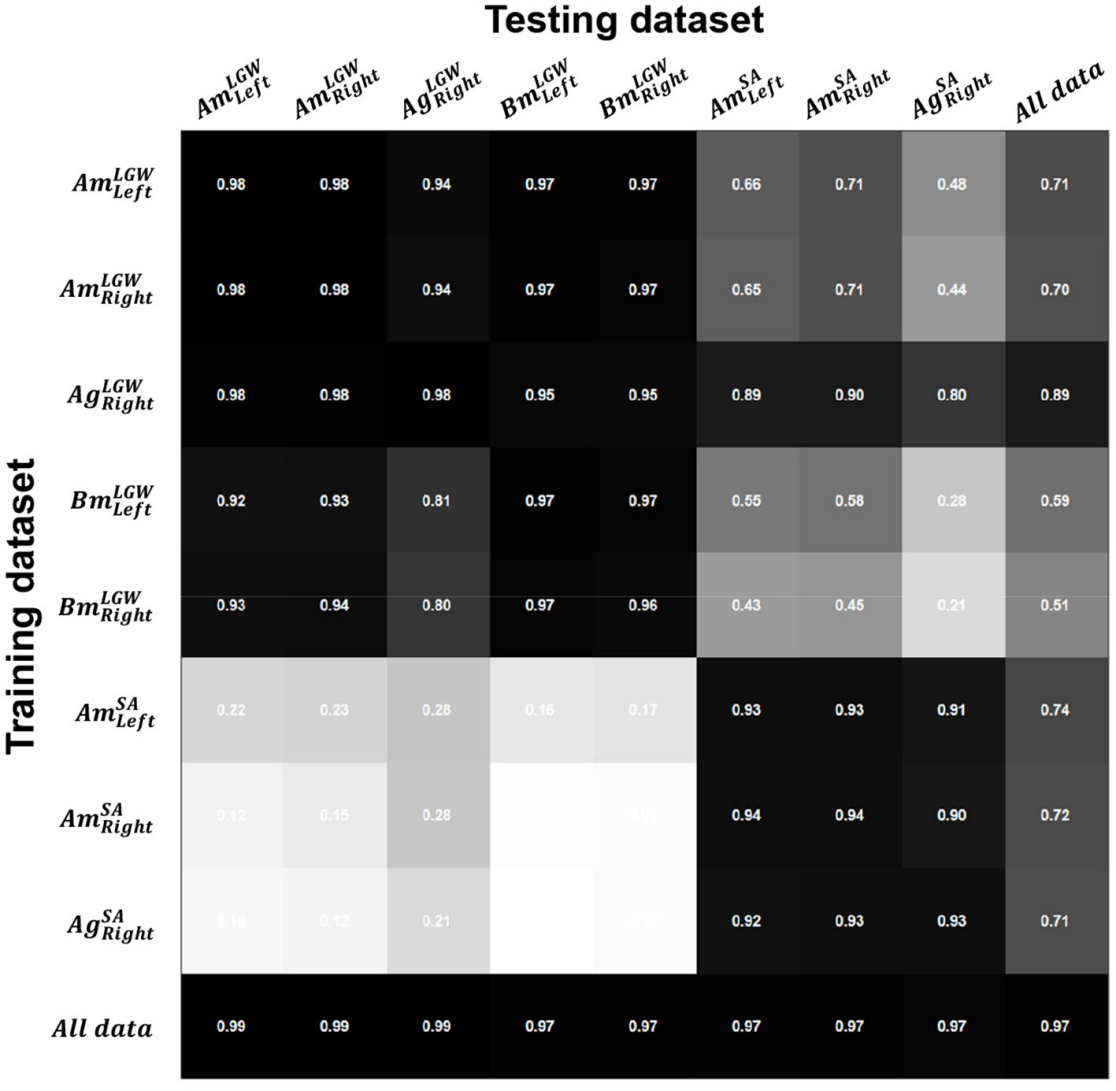
Coefficients of determination (*R*^2^) for the difference between the actual and predicted gait phases. Intra-mode prediction showed higher *R*^2^; on the other hand, inter-mode prediction (e.g., prediction of SA datasets using a model trained on an LGW dataset) showed poor performance owing to the different gait characteristics. The model trained on all datasets (i.e., the last row) showed consistent performance with an average *R*^2^ of 0.97 across all sub-divided datasets.

Figure 3 shows *R*^2^ for each dataset using each model. Since LGW and SA have different gait trajectories, they showed low performance when predicting each other (i.e., inter-mode prediction). However, the model trained on all benchmark datasets (i.e., the last row) showed consistent performance; the average *R*^2^ of 0.97 was obtained.

**Figure 4:**
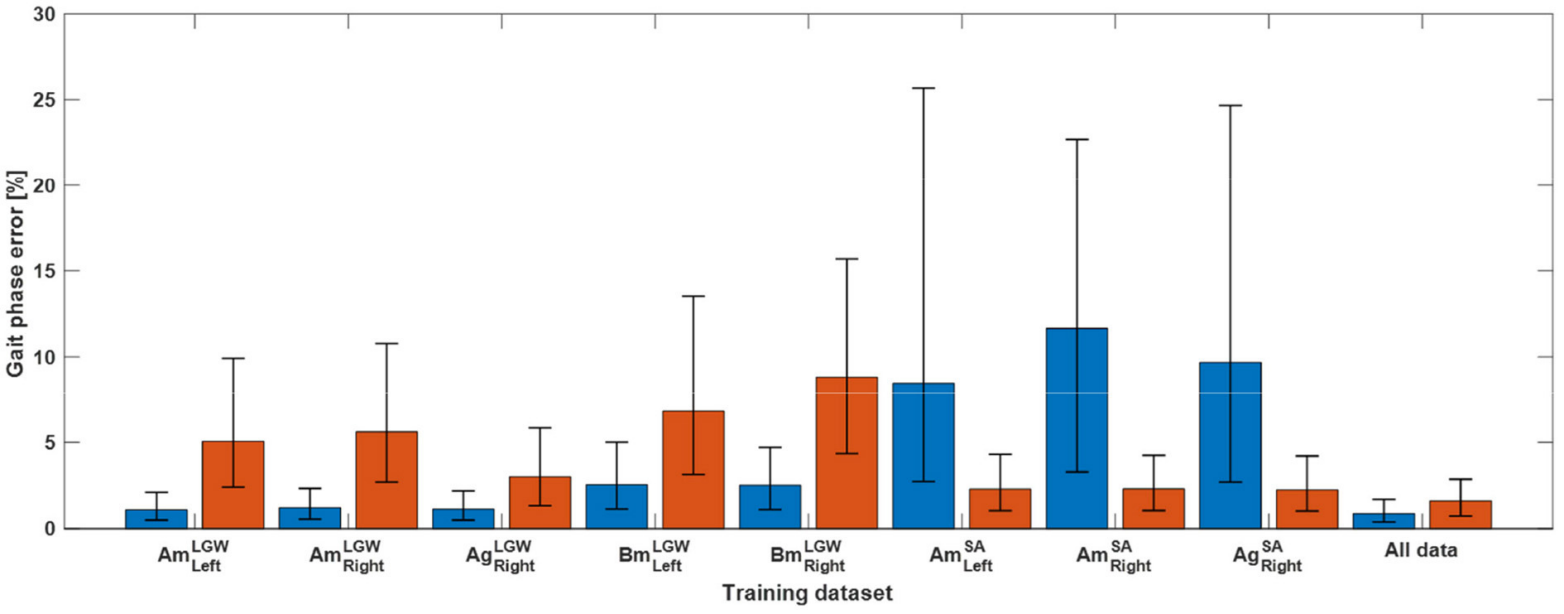
Gait phase prediction error. The blue and red bars indicate errors for five LGW and three SA datasets, respectively. Inter-mode prediction errors were higher than intra-mode prediction errors. The model trained using all datasets showed average prediction errors of 1.27% and 2.07% for LGW and SA, respectively; the average error of 1.28% across all ambulation modes was obtained.

The gait phase prediction error showed a similar trend (Figure 4). Intra-mode predictions had lower errors than inter-mode predictions. In particular, the models trained on SA datasets (i.e., $Am_{Left}^{SA}$, $Am_{Right}^{SA}$, and $Ag_{Right}^{SA}$) showed errors of more than 10% on the LGW datasets. On the other hand, the model trained on all benchmark datasets showed average prediction errors of 1.27% and 2.07% for LGW and SA, respectively.

**Figure 5:**
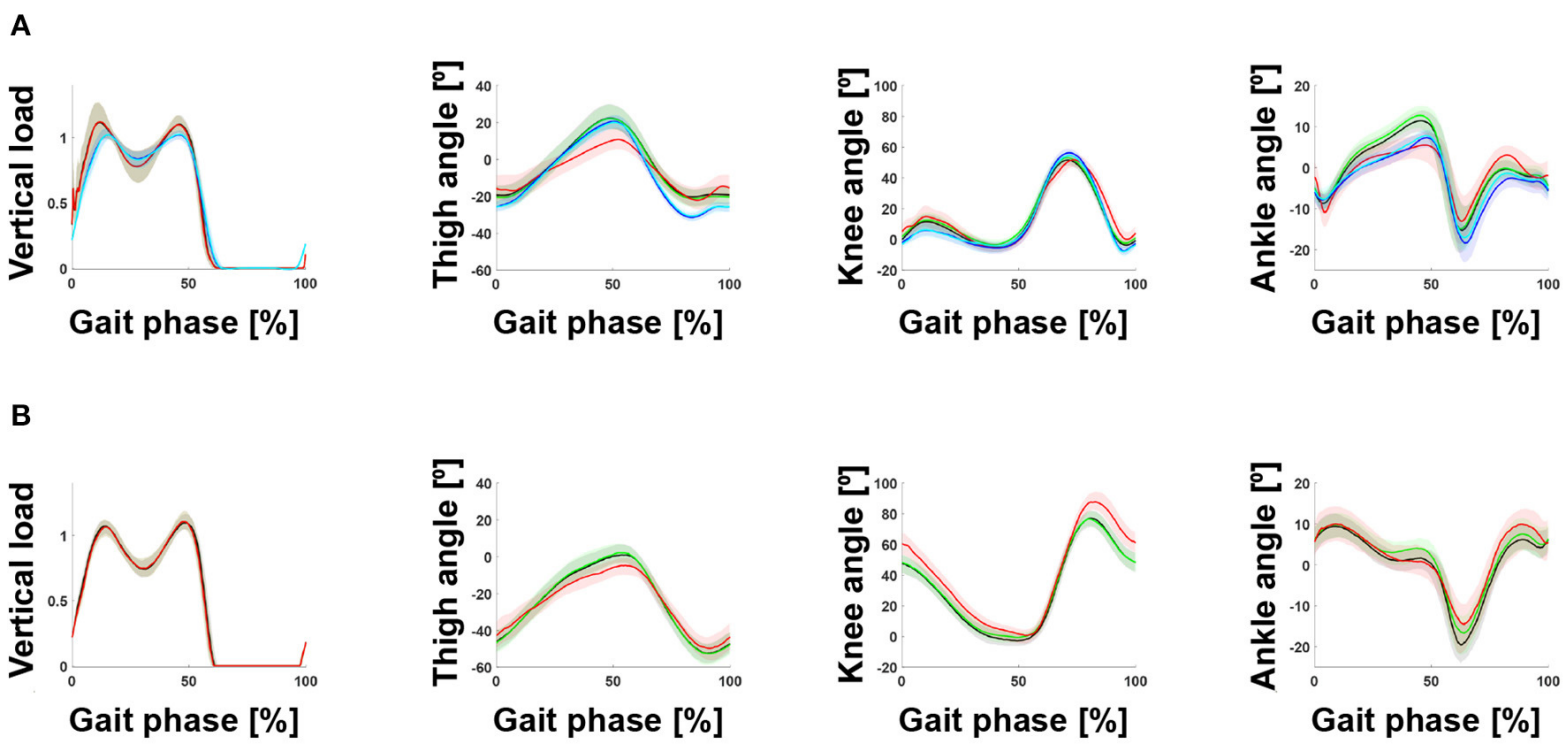
Gait trajectory from benchmark datasets. **(A)** The black, green, red, blue, and cyan lines indicate $Am_{Left}^{LGW}$, $Am_{Right}^{LGW}$, $Ag_{Right}^{LGW}$, $Bm_{Left}^{LGW}$, and $Bm_{Right}^{LGW}$, respectively. **(B)** The black, green, and red lines indicate $Am_{Left}^{SA}$, $Am_{Right}^{SA}$, and $Ag_{Right}^{SA}$, respectively. Although motion capture data and goniometer data (e.g., $Am_{Right}^{LGW}$ and $Ag_{Right}^{LGW}$) were measured simultaneously, a significant difference in joint angles was observed: The root mean square differences for the thigh, knee, and ankle angles were 6.2°, 4.3°, and 3.7° for LGW, and 4.3°, 8.3°, and 1.9° for SA, respectively. All plots show the 75th and 25th percentiles in lighter bands.

In the case of dataset A, although motion capture and goniometer data (e.g., $Am_{Right}^{LGW}$ and $Ag_{Right}^{LGW}$) were recorded simultaneously, the joint angles differed significantly (Figure 5). These differences in sensor configurations may have caused the differences in prediction performance. In particular, the goniometer data-based model was more robust than the motion capture data-based model (see second and third rows in Figure 3).

### 3.2. Evaluation of OSL data

**Figure 6:**
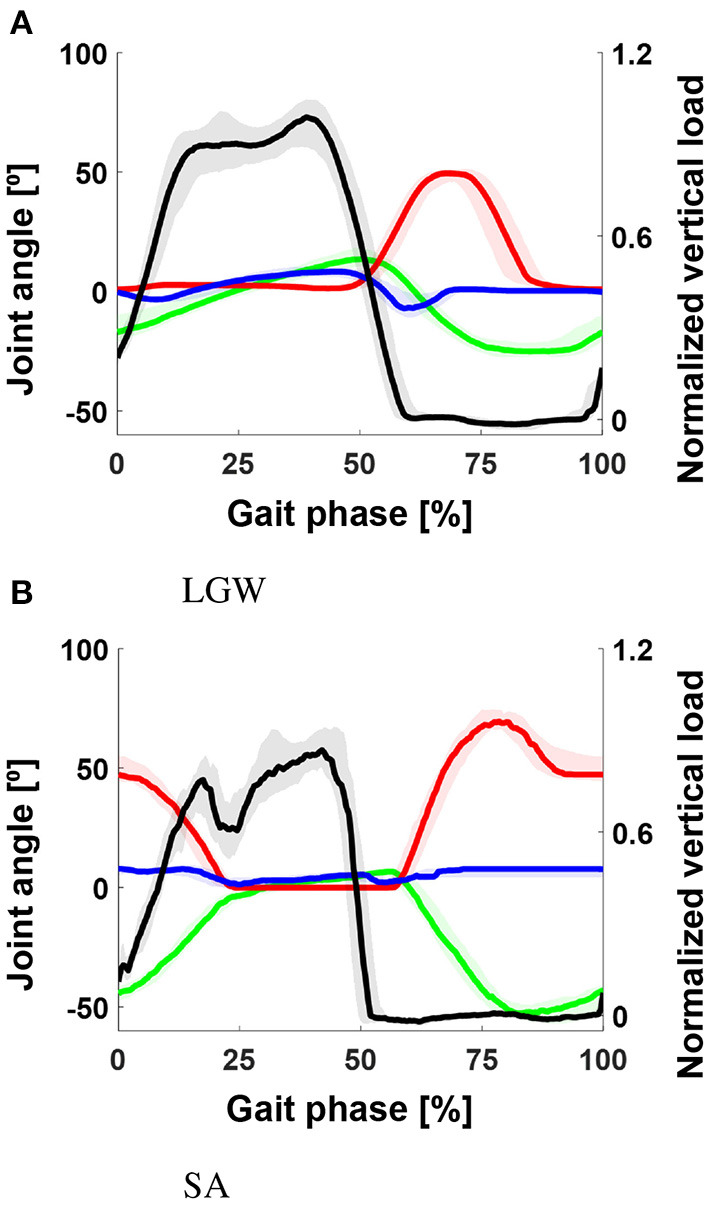
Gait trajectory for LGW **(A)** and SA **(B)** from four transfemoral amputee users. The black, green, red, and blue lines indicate the vertical load and thigh, knee, and ankle angles, respectively. The ankle trajectories for SA showed a significantly low correlation with the benchmark datasets (Figure 5). All plots show the 75th and 25th percentiles in lighter bands.

Figure 6 shows the gait trajectories of a state machine-controlled OSL from all four transfemoral amputee users. In the case of the LGW, the Pearson correlations with the vertical load, thigh, knee, and ankle angles in the benchmark datasets were 0.94, 0.94, 0.93, and 0.92, respectively. However, in the case of the SA, the correlations were 0.83, 0.96, 0.96, and 0.24, respectively; ankle angles showed a significantly low correlation.

**Figure 7:**
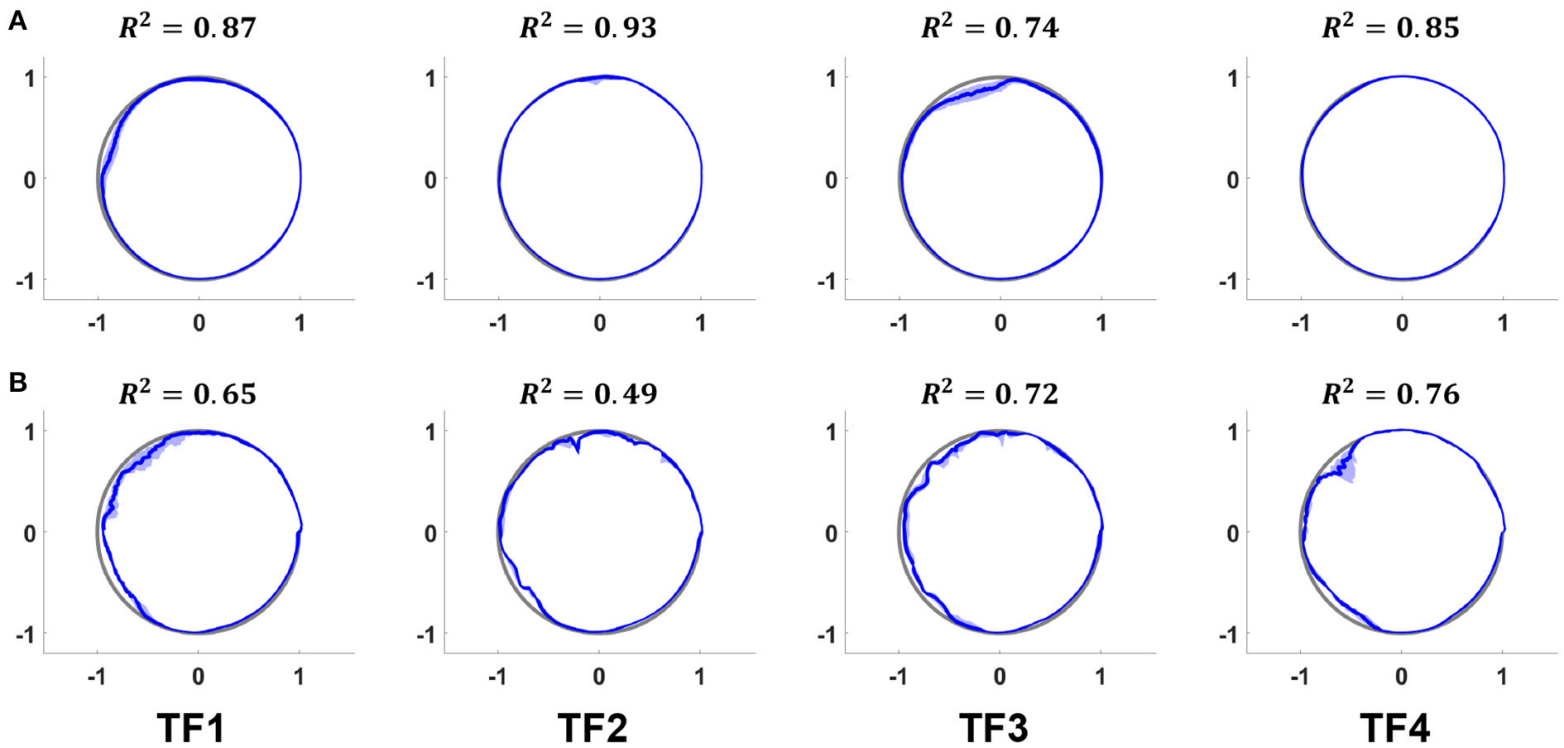
Actual (gray lines) and predicted (blue lines) phases for LGW **(A)** and SA **(B)** in the polar coordinate system. The average *R*^2^ for all users was 0.72. Although the prediction performance varies from user to user, most errors were observed in the late stance phase. All plots show the 75th and 25th percentiles in lighter bands.

**Figure 8:**
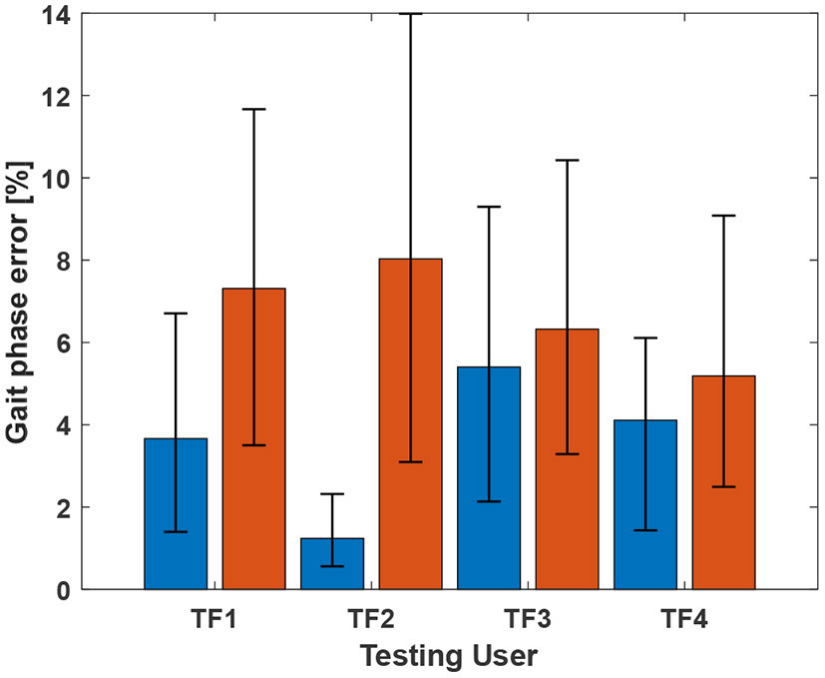
Gait phase prediction error. The blue and red bars indicate errors for LGW and SA, respectively. The average errors for LGW and SA were 4.82% and 8.06%, respectively. Error bar represents 25th and 75th percentiles.

The phase prediction performance (Figures 7, 8) varies from user to user; the average *R*^2^ for all users across all ambulation modes was 0.72, and differences were generally observed in the late stance phase. The gait phase error of SA (8.06%) was higher than that of LGW (4.82%); the average error across all ambulation modes was 5.70%.

## 4. Discussion

The proposed method can learn the continuous gait phase from sensor data (i.e., the vertical load and lower limb joint angles) commonly available in powered lower-limb assistive devices. The model was trained using benchmark datasets and applied to a powered prosthetic leg with a different sensor configuration without tuning. Sensor data, such as vertical loads and joint angles, may depend on sensor characteristics. Therefore, a model trained on a specific sensor dataset may have poor prediction performance when testing the model on a different sensor dataset. For example, in the case of dataset A, the joint angles were simultaneously collected using a motion capture system and goniometers. However, the joint trajectories (Figure 5) and the performance of the models (Figures 3, 4) based on them differed. These results demonstrate that a gait phase prediction model trained using a specific system may not be easily adapted to other systems. These limitations suggest that a device-agnostic approach is needed. The end-to-end capabilities of a deep neural network enable the integration of data from different datasets and the development of a unified device-agnostic model. As demonstrated in this work, applying a model trained from benchmark datasets to powered lower-limb assistive devices without additional tuning will help to validate hardware or software easily.

Eliminating discontinuity is a crucial factor in the DNN-based gait phase prediction. We tried to use the 1-D variables in the Cartesian coordinate system rather than the 2-D variables in the polar coordinate system as the output of the network. However, it degraded prediction performance compared to the 2-D variables with most errors made around 0% or 100%. An example case is described in the Supplementary Document S1.

Prediction performance may depend on the configuration of the DNN. In this study, 250 ms of sensor data history were used to formulate predictions. This parameter was chosen based on our previous work (Kim and Hargrove, 2022), in which 250 ms of vertical load and thigh angle history was best to predict desired impedance parameters.

We scaled input and output data for the proposed DNN using Equations (2) and (4). This scaling is not mandatory and there may be better scaling methods. The reason for the scaling is that the OSL, which is the prosthetic leg we use, transmits and receives data in the scaled values. We wanted to minimize additional process for data conversion by following the data scaling of the OSL for our future work, real-time evaluation gait phase and updating controllers.

In this study, we heuristically determined the network architecture hyperparameters including the number the layers, number of units in the layers, and activation functions. We adapted the number of units in dense layers from our previous work for controlling prosthetic legs using DNN (Kim and Hargrove, 2022). In the case of the activation function, sigmoid was better than other activation functions, such as tanh and ReLU. We speculate that the sigmoid function is suitable for our small range near 0.5 of input and output to the DNN. In addition, additional layers may improve the performance; however, execution time for prediction will be increased as a trade-off.

The LGW and SA models showed poor inter-mode prediction performance due to differences in gait trajectories. However, the LGW models showed slightly better inter-mode prediction performance than the SA models. This difference in performance may be a result of the amount of training data, as LGW datasets have more data than SA datasets; the models based on SA datasets had fewer variations and may have overfitted. This speculation is also supported by the fact that dataset A showed better performance than dataset B; dataset A has nearly 20 times more data than dataset B (see Table 1).

In addition, one noticeable prediction result is that the models trained using $Ag_{Right}^{LGW}$ showed better performance on SA datasets than other LGW datasets. We speculated that the large variation in the joint angles from the goniometers improved the robustness of the model. Thus, model training using a variety of datasets and different sensor configurations may improve the overall performance of gait phase prediction for untrained modes.

In general, the state machine parameters for the stance phase should be tuned to generate a more natural gait similar to non-disabled individuals. Specifically, the ankle parameters for SA should be improved. In details, the ankle trajectory (blue line, Figure 6B) is too flat compared to the non-amputee trajectory (Figure 5B). A controller should provide more dynamic movement in the mid-to-late stance phase. In conclusion, the model provided information on how to improve the controller in addition to the similarity between gait data from the benchmark datasets.

The proposed method has several limitations. First, it was limited to level-ground walking and ascending stairs. Additional ambulation modes should be considered, including turning and descending stairs and ramps. We believe use of additional benchmark data can overcome this limitation. For example, dataset A contains data for turning on the ground. Training with these data will prove the robustness of the phase prediction model. Furthermore, to ensure that the method can be applied in practice, the model must be able to correctly identify the ambulation mode from the sensor data (i.e., ambulation mode classification). Second, although improving the performance of the controller for a powered prosthetic leg may be possible, the focus of this study was on evaluating the pre-developed controller. For instance, control parameters can be updated in real-time to reduce differences in gait phase and trajectory. However, updating the controller is beyond the scope of this study and will be performed in future work.

Real-time gait phase prediction may significantly improve the gait by incorporating various control methods. For example, walking at various speeds and slopes was facilitated using virtual constraints based on a human-inspired phase variable (Quintero et al., 2018) for controlling a prosthetic leg.

To investigate feasibility, we deployed the proposed model on a smartphone running Android 12 (Galaxy Z Flip 3, Samsung). The Android parsed sensor data every 5 ms from the OSL. Execution time for the deployed network was approximately 1.2 ms. In conclusion, model predicted the gait phase using a 250 ms history of sensor data at intervals of 5 ms, and this prediction took approximately 1.2 ms. This indicates that the model has the potential to predict in real-time. The Supplementary Video S2 contains real-time gait prediction based on the sensor data from the OSL, for which an intact limb user performed LGW on the OSL using a bypass adaptor. Performance was reasonable, but a significant error was observed when the user turned in place to change the body direction, as turning was not in the training datasets. This result shows the limitation of the proposed method: poor performance when an untrained mode was performed. In future work, we will continue to improve the robustness of untrained ambulation modes on various terrains. Then, the model will be used in the real-time update of control parameters to generate a normal gait similar to that in training datasets.

The proposed DNN model outperformed general machine learning-based algorithms (see the Supplementary Document S2). However, obviously, the performance of machine learning algorithms depends on model parameters as DNN models do; a state-of-the-art machine learning method may outperform the proposed DNN model. Despite this limitation, this work demonstrated the feasibility of device-agnostic gait phase prediction and evaluation could be possible by analyzing the history of the sensor data commonly available on powered lower-limb assistive devices.

## 5. Conclusion

In this study, we propose a gait phase prediction method using a DNN. The predictions are based on a set of sensor data commonly available on powered lower-limb assistive devices. The model was trained using benchmark datasets containing gait data of intact-limb individuals and applied to evaluate the performance of a powered prosthetic leg. The model successfully predicted the gait phase of individuals with transfemoral amputation using a powered lower-limb prosthesis and provided information on the differences between intact limb individuals from the benchmark datasets.

Hypothesis (i) was partially confirmed because performance may depend on the characteristics of the sensors and the amount of data used for training. In addition, the proposed model did not require tuning to evaluate the gait of transfemoral amputee patients wearing a powered prosthetic leg. Furthermore, it provided information on the differences between the actual gait and testing data, confirming Hypothesis (ii).

## Data availability statement

The original contributions presented in the study are included in the article/Supplementary material, further inquiries can be directed to the corresponding author/s.

## Ethics statement

The studies involving human participants were reviewed and approved by Northwestern University Institutional Review Board. The patients/participants provided their written informed consent to participate in this study.

## Author contributions

MK designed the study, performed data collection, analyzed data, and wrote the first draft. LH supervised and administered the project and revised the manuscript. Both authors contributed to the article and approved the submitted version.

## Supplementary material

Supplementary Video 1 S2 Gait phase extraction.

Supplementary Video 2 S3 Real-time gait phase prediction.

Supplementary Document 1 S1 Performance comparison between prediction of 1-D and 2-D gait phase variables.

Supplementary Document 2 S4 Performance comparison with general machine learning methods.
