# Supplementary material for: A gait phase prediction model trained on benchmark datasets for evaluating a controller for prosthetic legs
Source: Front Neurorobot. 2023 Jan 5;16:1064313. doi: 10.3389/fnbot.2022.1064313 (PMC9849563; doi:10.3389/fnbot.2022.1064313)
Supplement: Supplementary Document 1 — S1 Performance comparison between prediction of 1-D and 2-D gait phase variables. [file Data_Sheet_1.PDF]

S1. Performance comparison between prediction of 1-D and 2-D gait phase variables

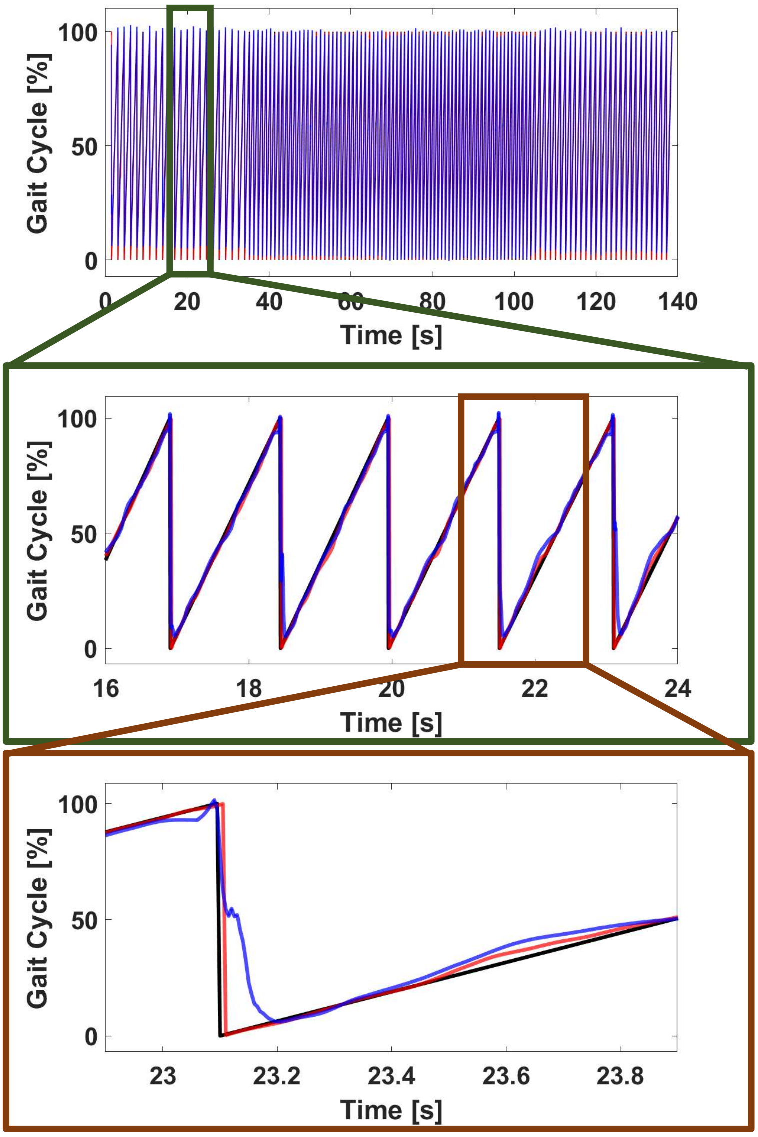

DNN models were trained on Am_{Left}^{LGW}. Both models have the same structure except for the output layers. Then, prediction was performed on data *Left_treadmill_01_01* of $AB06$. Black, red, and blue lines indicate the actual gait phase, gait phase predicted based on the 2-D variable, and gait phase based on the 1-D variable, respectively. 2-D and 1-D variable-based prediction showed median prediction errors of 0.74% and 1.39%, respectively.
