# Supplementary material for: A gait phase prediction model trained on benchmark datasets for evaluating a controller for prosthetic legs
Source: Front Neurorobot. 2023 Jan 5;16:1064313. doi: 10.3389/fnbot.2022.1064313 (PMC9849563; doi:10.3389/fnbot.2022.1064313)
Supplement: Supplementary Document 2 — S4 Performance comparison with general machine learning methods. [file Data_Sheet_2.pdf]

S4. Performance comparison with general machine learning methods

General machine learning algorithms trained on a training dataset (Am^{LGW}_{Right} and Am^{SA}_{Right}) were used to estimate the gait phase on a different dataset containing Ag^{LGW}_{Right} and Ag^{SA}_{Right}. We used MATLAB 2022b built-in functions to train the models; regression tree ensemble (RTE), gaussian process regression (GPR), linear regression (LR), and support vector machine regression (SVMR) models were trained using *fitrensemble*, *fitrgp*, *fitlm*, and *fitrsvm*, respectively. We chose these algorithms because LR is the most common regression algorithm, and RTE, GPR, and SVMR models are generally used for gait analysis [1, 2, 3].

Input and output data were the same as the proposed DNN model; we just flattened the input data (i.e., $R^{50\times4}$ to R^{200}). Generally, we used default model parameters. However, the maximum number of iterations for the GPR and SVMR models was limited to 100 and 10,000, respectively, because the models were not converged. In addition, in the case of the GPR model, the dimension of input data was reduced to 10, which satisfied the reduction criterion of 99%, using principal component analysis; processing with the original input data took too much time. The training time for the proposed DNN, RTE, GPR, LR, and SVMR models was 1 h, 12 h, 6 h, 0.1 h, and 6 h, respectively.

For the evaluation of the models, 10% of input-output pairs were randomly extracted for each dataset; there were more than 4,500,000 input-output pairs in total for each dataset. The gait phase estimation result is shown in the figure below.

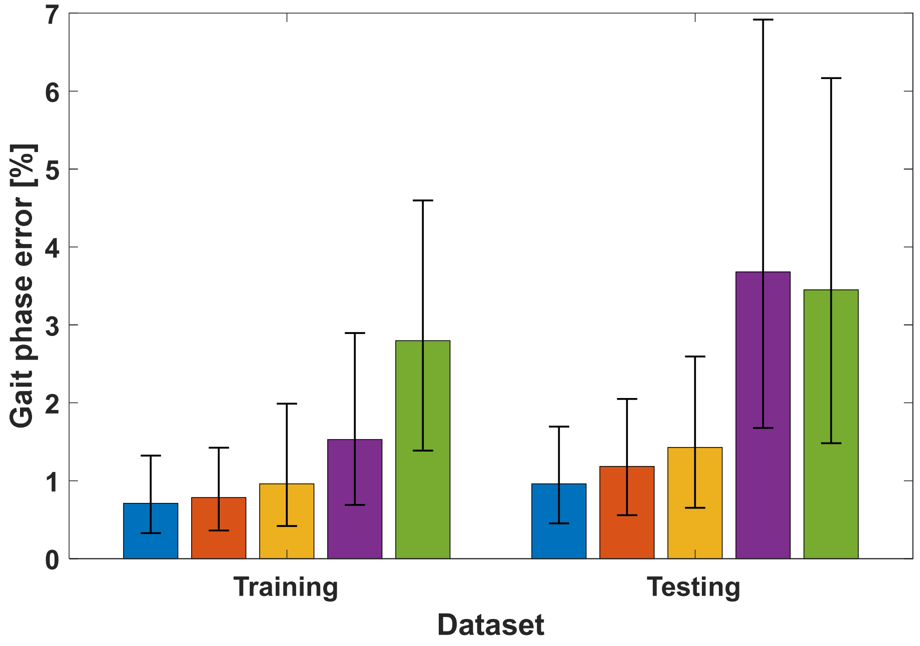

Gait phase prediction error. The blue, red, yellow, purple, and green bars indicate errors for the proposed DNN, RTE, GPR, LR, SVMR, respectively. Error bar represents 25th, 50th and 75th percentiles. The median error for the training dataset (i.e., Am^{LGW}_{Right} and Am^{SA}_{Right}) was 0.71 %, 0.78 %, 0.96 %, 1.53 %, and 2.80 % for DNN, RTE, GPR, LR, and SVMR, respectively; the median error for the testing dataset (i.e., Ag^{LGW}_{Right} and Ag^{SA}_{Right}) was 0.96 %, 1.18%, 1.43 %, 3.68 %, and 3.45 % for DNN, RTE, GPR, LR, and SVMR, respectively. RTE showed reasonable performance, albeit with significant differences ($p < 0.005$) from the DNN.

References

[1] R. K. Begg, M. Palaniswami, and B. Owen, "Support vector machines for automated gait classification," *IEEE transactions on Biomedical Engineering*, vol. 52, no. 5, pp. 828–838, 2005.

[2] A. Gupta and V. B. Semwal, "Multiple task human gait analysis and identification: ensemble learning approach," in *Emotion and information processing.* Springer, 2020, pp. 185–197.

[3] Y. Yun, H.-C. Kim, S. Y. Shin, J. Lee, A. D. Deshpande, and C. Kim, "Statistical method for prediction of gait kinematics with gaussian process regression," *Journal of biomechanics*, vol. 47, no. 1, pp. 186–192, 2014.
